# Granulocytic Myeloid‐Derived Suppressor Cells Promote the Stemness of Colorectal Cancer Cells through Exosomal S100A9

**DOI:** 10.1002/advs.201901278

**Published:** 2019-07-22

**Authors:** Yungang Wang, Kai Yin, Jie Tian, Xueli Xia, Jie Ma, Xinyi Tang, Huaxi Xu, Shengjun Wang

**Affiliations:** ^1^ Department of Laboratory Medicine The Affiliated People's Hospital Jiangsu University Zhenjiang 212002 China; ^2^ Department of Immunology Jiangsu Key Laboratory of Laboratory Medicine School of Medicine Jiangsu University Zhenjiang 212013 China; ^3^ Department of Laboratory Medicine The First People's Hospital of Yancheng City Yancheng 224000 China; ^4^ Department of General Surgery Affiliated Hospital of Jiangsu University Zhenjiang 212001 Jiangsu China

**Keywords:** colorectal cancer, exosomes, myeloid‐derived suppressor cells, S100A9, stemness

## Abstract

Cancer stem cells play a critical role in colorectal cancer (CRC) progression. Myeloid‐derived suppressor cells (MDSCs) promote tumor progression through multiple mechanisms in CRC. The roles of MDSCs in CRC cell stemness are unclear. MDSC‐derived exosomes are proposed to act as intercellular messengers. Herein, it is reported that granulocytic MDSCs (G‐MDSCs) promote CRC cell stemness and progression in mice through exosomes. It is found that S100A9, is highly expressed in G‐MDSC‐derived exosomes, and its blockade suppresses CRC cell stemness and the susceptibility of mice to AOM/DSS‐induced colitis‐associated colon cancer. Hypoxia induces G‐MDSCs to secrete more exosomes in a hypoxia‐inducible factor 1α (HIF‐1α)‐dependent manner, and respiratory hyperoxia can reduce CRC cells stemness through the inhibition of GM‐Exo production. Study‐based CRC patients also show that human MDSCs enhance CRC cell stemness and growth via exosomal S100A9, and plasma exosomal S100A9 level in CRC patients is markedly higher than that in healthy subjects. Thus, this study suggests that G‐MDSCs promote CRC cell stemness and growth through exosomal S100A9. Moreover, respiratory hyperoxia may be a beneficial strategy to reduce CRC cells stemness through the inhibition of GM‐Exo production. MDSCs exosomal S100A9 may be a marker for predicting the development of CRC.

## 1 Introduction

Colorectal cancer (CRC) is the most common malignant digestive tumor. Large‐scale efforts to explore the pathogenic mechanisms and diagnostic molecules associated with this disease need to be developed.^1^ Cancer stem cells (CSCs) are cancer cells that possess characteristics associated with normal stem cells, specifically the ability to self‐renew and differentiate into multiple cell types.^2^ CSCs are considered key cells in malignancy and therapeutic targets in CRC,^3^, ^4^ but the factors that influence the generation of these stem cells remain unclear. Extrinsic factors provided by the tumor microenvironment may be essential for promoting the stemness of CRC cells.^5^, ^6^ MDSCs originate from myeloid progenitors and are expanded and activated substantially in cancer.^7^, ^8^, ^9^ Two main MDSC populations have been characterized: monocytic MDSCs (M‐MDSCs) and granulocytic (also called polymorphonuclear) MDSCs (G‐MDSCs or PMN‐MDSCs). G‐MDSCs are the prevalent population of MDSCs.^10^ MDSCs are essential to promote the progression in CRC patients and experimental animal model through mediating immune suppression, angiogenesis, and epithelial‐mesenchymal transition (EMT).^7^, ^11^ The number of MDSCs in the blood correlates well with the clinical cancer stage and metastatic burden in CRC patients.^12^ Recently, MDSCs identified as a modulator of tumor cell stemness. MDSCs enhance the stemness of ovarian cancer cells by triggering microRNA101 expression in cancer cells and subsequently repress the corepressor gene C‐terminal binding protein‐2 (CtBP2).^13^ MDSCs endow stem‐like qualities to breast cancer cells through IL‐6/STAT3 and NO/NOTCH signaling.^14^ However, the effect of MDSC on CRC cells stemness is unclear. Therefore, a better investigation of the relationship between MDSC and CRC cells stemness is need in order to improve CRC intervention.

In multicellular organisms, cells exchange information by sending out single molecules or extracellular vesicles (EVs), which are also known as exosomes and microvesicles (MVs). Exosomes are 30–150 nm membrane vesicles that are released via fusion with the cell membrane.^15^ Exosomes consist of a lipid bilayer membrane surrounding a small cytosol and are devoid of cellular organelles. Exosomes have been proposed to act as intercellular communicators between sender cells and receiver cells via complex cargo, including proteins, lipids, and nucleic acids.^16^ Researchers have elucidated the complex roles of exosomes in cancer progression.^17^ Exosomes from murine MDSCs carry biologically active molecules^18^ and play important roles in cancer progression.^19^ Especially, S100A9 was abundant in MDSC‐ derived exosomes.^18^ Although S100A9 was previously shown to up‐regulated in colorectal cancer, the relationship between MDSC exosomal S100A9 and colorectal cancer is unclear. Our previous work showed that G‐MDSC exosomes (GM‐Exo) could inhibit Th1 cell proliferation and promote Treg expansion in mice with dextran sulfate sodium (DSS)‐induced colitis.^20^ These results drove us to further observe whether MDSCs communicate with CRC cells via exosomes secretion.

We set out to characterize the role of GM‐Exo in the stemness of CRC cells and seek the main component involved using colon carcinoma cell lines and mice with CRC. Then, we explored the effect of hypoxia on GM‐Exo secretion and the effect of respiratory hyperoxia on the inhibition of GM‐Exo secretion and CRC cells stemness. Last, we detected the level of plasma exosomal S100A9 in different CRC patients. Here, we present for the first time that G‐MDSCs enhance CRC cell stemness via exosomes secretion and S100A9 is the crucial component that mediate GM‐Exo‐promoted CRC cell stemness. We demonstrate that hypoxia plays a key role in exosomes secretion from G‐MDSCs through promoting Rab27a expression and respiratory hyperoxia reduces colon cancer cells stemness through inhibiting GM‐Exo production. Consistent with these results, human MDSC exosomal S100A9 promote human CRC cells stemness and the level of S100A9 is elevated in plasma exosomes isolated from CRC patients. In summary, our study describes a previously unknown pro‐CRC circuit through which G‐MDSC exosomal S100A9 can induce the stemness of CRC cells that foster the development of CRC. G‐MDSC exosomal S100A9 may become a good therapeutic target for CRC patients. Respiratory hyperoxia could reduce colon cancer cells stemness through inhibiting GM‐Exo production. The changes in plasma exosomal S100A9 could provide clues concerning CRC occurrence and recurrence, as well as a functional component of CRC cell stemness.

## 2 Results

### 2.1 G‐MDSCs Enhance the Stemness of Colon Cancer Cells via Exosomes

**Figure 1:**
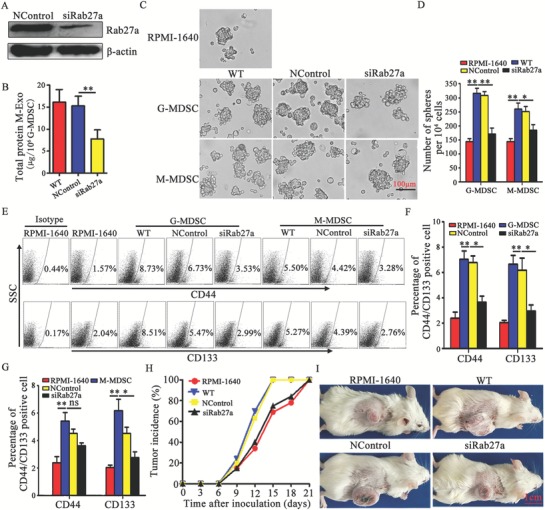
MDSCs enhance the stemness of colon cancer cells by exosomes secretion. A) Representative western blot analysis of Rab27a expression in total MDSCs and Rab27a siRNA‐treated total MDSCs (representative of three independent experiments). NControl: negative control. B) Measurement of total protein in exosomes secreted by total MDSCs. C,D) Effects of MDSC subpopulations on cancer sphere formation. Primary CT‐26 cells and MDSC subpopulations were cocultured under sphere‐forming conditions for 14 days. A sphere formation assay was performed. Representative photographs were taken (C), and the number of spheres (D) with a diameter >75 µm was counted under a light microscope. E–G) Representative percentages of CD44^+^ or CD133^+^ cells after the coculture of MDSC subpopulations and CT‐26 cells were detected by FCM (E,G), and the results were analyzed statistically (F). H,I) The effect of G‐MDSCs on the development of a CT‐26 tumor model. Tumor‐bearing mice were treated with 1 × 10^6^ G‐MDSCs or Rab27a siRNA‐transfected G‐MDSCs. The tumor incidence was observed every three days (H). A representative tumor from each group is shown on day 21 (I). In B,D,FG) data are shown from three independent experiments. *p < 0.05 and **p < 0.01, analyzed by ANOVA.

Ras‐related RAB proteins control exosomes biogenesis and secretion,^21^, ^22^ and Rab27a is a key molecule in exosomal pathway.^23^ We reasoned that inactivating this gene would reduce exosomes production by MDSCs. The Rab27a isoform was selected for knockdown with a specific siRNA in total MDSCs. The expression of Rab27a was effectively reduced (**Figure** **1**A), and exosomes secretion was obviously decreased after the MDSCs were treated with siRNA‐Rab27a (Figure 1B). To determine whether MDSC subpopulations promote the stemness of colon cancer cells through exosomes secretion, G‐MDSCs or M‐MDSCs were treated with siRNA‐Rab27a and then co‐cultured with CT‐26 cells. A tumor sphere formation assay was carried out, and the levels of a panel of established CSC markers, including CD133 and CD44, were analyzed. As shown in Figure 1C–G, G‐MDSCs and M‐MDSCs promoted tumor sphere formation and increased the CD44^+^ and CD133^+^ cell percentages. However, the tumor sphere numbers and CD133^+^ cell percentages were decreased when exosomes were inhibited in G‐MDSCs and M‐MDSCs, but the CD44^+^ cell percentage was decreased only when exosomes were inhibited in G‐MDSCs (Figure 1C–G). These results suggest that G‐MDSCs and M‐MDSCs enhance colon cancer cell stemness in a manner partially dependent on exosomes secretion. Considering that G‐MDSCs are a dominant subpopulation of MDSCs, G‐MDSCs were selected to further observe the effect of exosomes. CT‐26 cell tumor‐bearing mice were adoptively transferred with siRNA‐Rab27a‐treated G‐MDSCs, and the tumor incidence and progression were observed. The results showed that the tumor incidence was lower and the tumor growth was slower in the siRNA‐Rab27a‐treated G‐MDSC group (Figure 1H,I). All these results suggest that MDSCs can enhance colon cancer cell stemness in a manner partially dependent on exosomes.

### 2.2 GM‐Exo Directly Promote the Growth of Colon Cancer Cells

Based on the exosomes production results, G‐MDSCs promoted colon cancer cell stemness. We attempted to investigate the direct effects of GM‐Exo on CT26 cells. When the tumor was greater than 2 cm in diameter, G‐MDSCs were isolated from CT26 cell tumor‐bearing mice, and the purity was more than 95% (Figure S1A, Supporting Information). G‐MDSCs could inhibit CD4^+^ T cell proliferation and IFN‐γ production, whereas normal neutrophils did not exhibit these effects (Figure S1D,E, Supporting Information). Membrane molecules and imaging by transmission electron microscopy are widely used for the characterization of exosomes. GM‐Exo displayed closed round vesicles with diameters of ≈100 nm (Figure S1F, Supporting Information). GM‐Exo expressed CD63 and CD9, which are characteristic exosomes molecules (Figure S1G, Supporting Information). In contrast, calnexin was not detected in the purified GM‐Exo preparations (Figure S1G, Supporting Information), indicating that GM‐Exo are free from contamination with nonexosomes membrane proteins. In addition, GM‐Exo could inhibit CD4^+^ T cell proliferation and IFN‐γ production, whereas neutrophil‐derived exosomes (Neu‐Exo) did not exhibit these effects (Figure S1H,I, Supporting Information).

**Figure 2:**
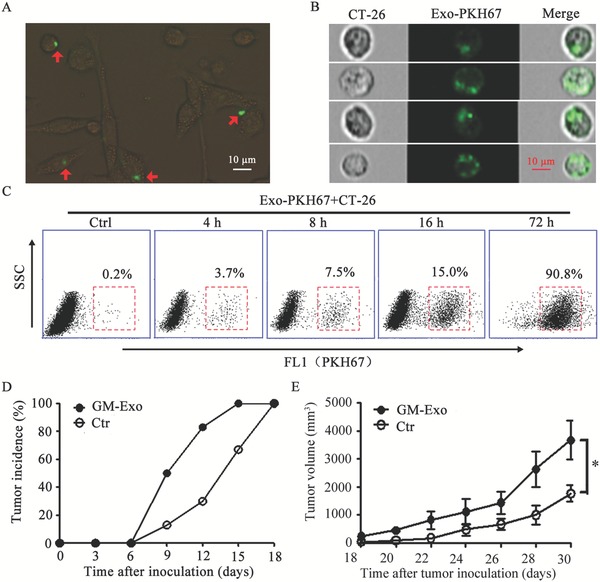
GM‐Exo directly promotes the growth of CT‐26 cells. A) The GM‐Exo distribution (green) in CT‐26 cells was analyzed by fluorescence microscopy, representative image of three independent experiments. B) The uptake of GM‐Exo by CT‐26 cells was identified with imaging flow cytometers. C) FCM analysis of PKH67‐exosomes‐positive cells. FITC‐positive cells were acquired on a FACS Calibur system, and the percentages of exosomes‐positive cells were quantified. In A–C) GM‐Exo (10 µg mL^−1^) were labeled with the lipophilic PKH67 dye (green) and added to the CT‐26 cultivation system for different time periods. Representative data were pooled from two independent experiments, *n* = 6. D,E) The effect of GM‐Exo on the development of CT‐26 cell‐bearing mice. The mice were injected with 1 × 10^6^ CT‐26 cells, which were pretreated with 10 µg mL^−1^ GM‐Exo for 72 h. The tumor incidence was observed every day (D). The tumor volume was determined every 3 days (E). Data are shown as the mean ± SEM of each group (*n* = 6) pooled from three independent experiments. *p < 0.05, analyzed by ANOVA.

For exosomes‐tracking purposes, purified GM‐Exo was labeled using the PKH67 fluorescent membrane dye. Images were obtained for exosomes‐positive cells. As shown in **Figure** **2**A,B, the green fluorescence‐labeled exosomes localize inside of CT‐26 cells. Moreover, the percentages of GM‐Exo fluorescence‐positive CT‐26 cells were increased with a prolonged effect time (Figure 2C). These data demonstrate that GM‐Exo could effectively bind to CT‐26 cells and then be internalized. We next investigated the effect of GM‐Exo on the growth of CT‐26 cells in vivo. BALB/c mice were subcutaneously injected into the right flank with 1 × 10^6^ CT‐26 cells treated with GM‐Exo for 72 h. The results showed that the tumor nodules in the GM‐Exo‐treated CT‐26 cell group were detected earlier (Figure 2D) and grew more rapidly than those in the control group (Figure 2E). These data confirm that GM‐Exo directly promotes the growth of colon cancer cells.

### 2.3 GM‐Exo Promotes the Stemness of Colon Cancer Cells through S100A9

**Figure 3:**
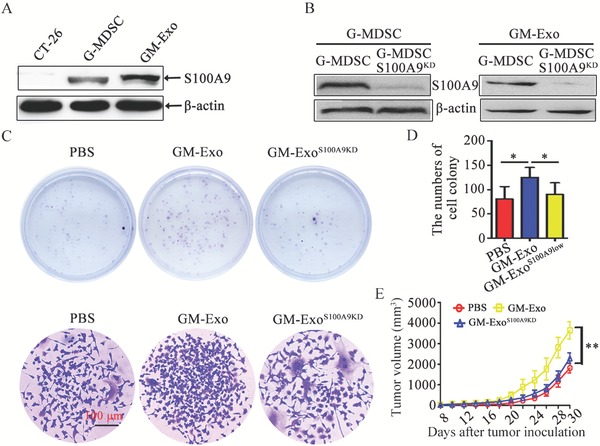
GM‐Exo promotes CT‐26 cell growth through S100A9. A) Representative western blot of S100A9 expression in GM‐Exo. B) Representative western blot analysis of S100A9 expression in G‐MDSCs and GM‐Exo treated with S100A9 siRNA or left untreated (representative result of three independent experiments). C) Representative images of cell colonies (Top) and a single colony (Bottom) in CT‐26 cells treated with PBS, GM‐Exo, or GM‐Exo^S100A9KD^ for 14 days. D) Colonies with more than 100 cells were quantified. E) The tumor volume was determined every 2 days. CT‐26 cells were treated with PBS, GM‐Exo, or GM‐Exo^S100A9KD^ for 14 days, and 1 × 10^6^ cells were subcutaneously injected into the right flank of BALB/c mice. The tumor length (*L*) and width (*W*) were measured with a caliper, and tumor volumes were calculated. Data are shown as the mean ± SEM of each group (*n* = 6) pooled from three independent experiments. *p < 0.05 and **p < 0.01, analyzed by ANOVA.

The above experimental results showed that GM‐Exo enhanced the stemness of colon cancer cells. To gain insight into the mechanisms through which GM‐Exo promote colon cancer cell stemness and subsequent tumor progression, we mined MDSC exosomes mass spectrometry data for potential stemness mediators.^18^ The pro‐inflammatory protein S100A9 was previously shown to be abundant in MDSC‐ derived exosomes and to have a chemotactic function for MDSCs.^18^, ^24^ As shown in **Figure** **3**A, abundant S100A9 protein was detected in GM‐Exo. Then, S100A9 expression was knocked down in G‐MDSCs using siRNA. GM‐Exo^S100A9KD^, which had lower expression of S100A9 in exosomes, was isolated and purified (Figure 3B). Colony formation was significantly increased in GM‐Exo‐treated CT‐26 cells, but this effect was markedly reduced in GM‐Exo^S100A9KD^‐treated cells (Figure 3C,D). Consistent with this phenomenon, tumor progression was significantly accelerated in mice injected with GM‐Exo‐treated CT‐26 cells compared with that in GM‐Exo^S100A9KD^‐treated CT‐26 cells (Figure 3E). These findings suggest that GM‐Exo promote the proliferation of colon cancer cells through S100A9.

**Figure 4:**
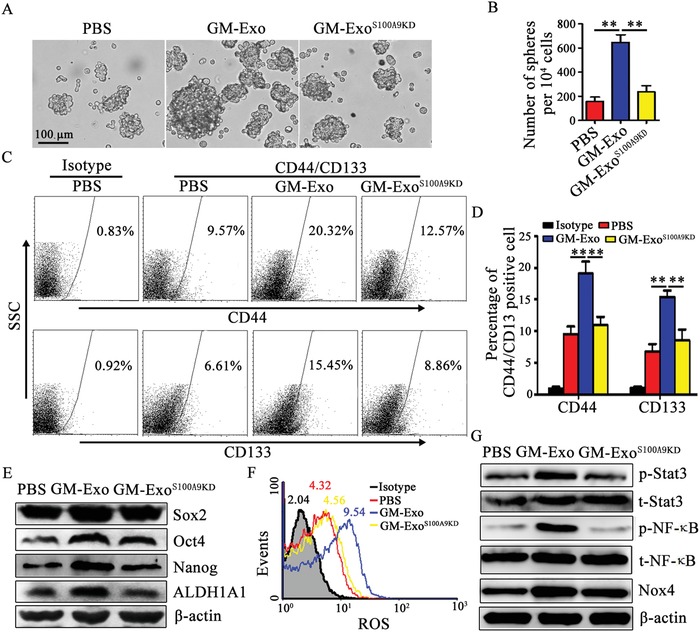
Exosomal S100A9 from G‐MDSCs enhances colon cancer cell stemness. A,B) GM‐Exo promoted colon cancer cell sphere formation through S100A9. In the presence of 10 µg mL^−1^ GM‐Exo or GM‐Exo^S100A9KD^, CT‐26 cells were subjected to the tumor sphere formation assay under sphere‐forming conditions for 14 days. Representative photographs were taken (A), and the number of spheres (B) with a diameter of >75 µm was counted. C,D) Representative percentages of CD44^+^ or CD133^+^ cells after 10 µg mL^−1^ exosomes treatment were detected by FCM (C), and the results were analyzed statistically (D). E) Western blot analysis was used to determine Sox2, Oct4, Nanog, and ALDH1A1 levels after treatment, as indicated. Representative results from three independent experiments. F) ROS levels in CT‐26 cells. CT‐26 cells were treated with GM‐Exo, and ROS levels were detected by FCM. G) Western blot analysis was used to determine p‐STAT3, STAT3, NF‐κB, p‐p65, and Nox4 levels after treatment, as indicated. Representative results from three independent experiments. In B,D) data are shown from three independent experiments. **p < 0.01, analyzed by ANOVA.

Next, we observed the role of S100A9 in the GM‐Exo‐mediated promotion of stemness and investigated the key molecules involved in this process. The number of tumor spheres, the percentages of CD44^+^ cells and CD133^+^ cells, and the expression of multiple stem cell core proteins (Sox2, Oct4, Nanog, and ALDH1A1) were all increased when the cells were treated with GM‐Exo but were markedly reduced in GM‐Exo^S100A9KD^‐treated CT‐26 cells (**Figure** **4**A–E). These data demonstrate that GM‐Exo enhance the stemness of colon cancer cells through S100A9.

S100A9 directly binds to components of the NADPH oxidase complex in MDSCs and potentiates the activation of NADPH oxidase, which causes increased production of reactive oxygen species (ROS).^25^ It has been reported that Nox/ROS can induce NF‐κB and STAT3 activation and promote the stemness of pancreatic cancer cells.^26^ Persistently activated STAT3 and NF‐κB in tumor cells act as crucial oncogenic mediators and promote cancer cell stemness and tumorigenesis.^26^, ^27^ Our results show that ROS and Nox4 levels in GM‐Exo‐treated colon cancer cells were increased, but ROS and Nox4 returned to their original levels in GM‐Exo^S100A9KD^‐treated colon cancer cells (Figure 4F,G). Furthermore, the phosphorylation levels of STAT3 and NF‐κB p65 were enhanced in GM‐Exo‐treated colon cancer cells, but the levels of p‐STAT3 and p‐p65 were decreased in GM‐Exo subjected to S100A9 knockdown (Figure 4G). Together, these results suggest that exosomal S100A9 from G‐MDSCs enhances the stemness of colon cancer cells.

### 2.4 Exosomal S100A9 from G‐MDSCs Enhances Susceptibility to AOM/DSS‐ Induced Colitis‐Associated Colon Cancer in Mice

The above results confirm that G‐MDSCs promote the stemness of colon cancer cells via exosomal S100A9. We observed the regulatory effects of exosomal S100A9 from G‐MDSCs on CRC cell stemness in a murine colitis‐associated colon cancer (CAC) model. Exogenous GM‐Exo was injected into CAC mice, and their biodistribution in colorectal tissues was detected. The results showed that GM‐Exo was more frequent in intratumoral tissues than in peritumoral tissues (Figure S2A, Supporting Information). Moreover, there was an increase in the GM‐Exo distribution after three injections compared to that after one injection (Figure S2A, Supporting Information). GM‐Exo uptake was more efficient in intratumoral tissue, and there was an increase in GM‐Exo uptake after three injections (Figure S2B, Supporting Information). Therefore, exogenous GM‐Exo is distributed in colorectal tissues and captured by colorectal tissue cells.

**Figure 5:**
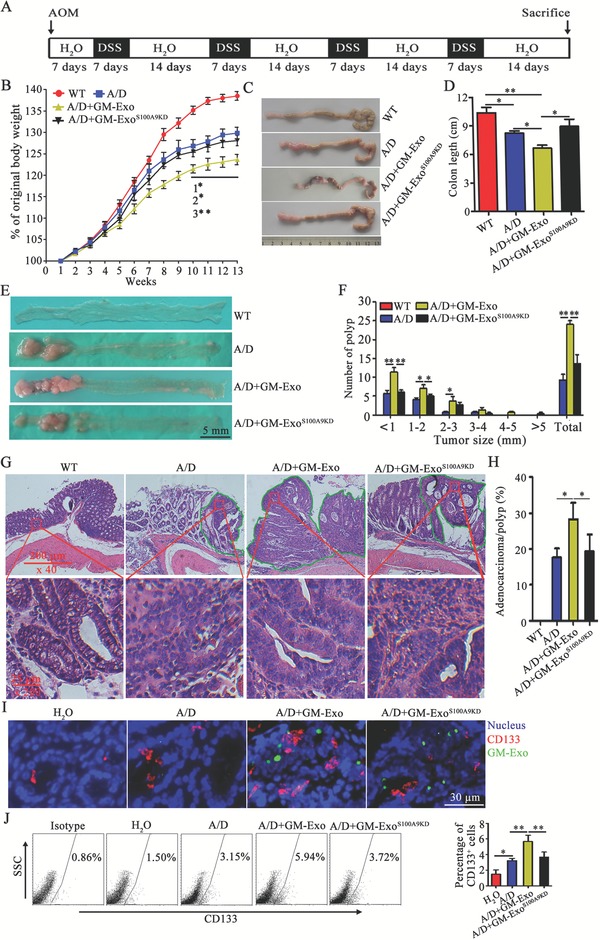
Exosomal S100A9 from G‐MDSCs enhances the susceptibility of mice to AOM/DSS‐induced CAC. A) Schematic of CAC mouse induction. B) Weekly changes in the body weights of mice in different groups. C) Representative examples of colorectal specimens from mice in different groups. D) The colorectal lengths were measured at the end of the experiments. E) Gross view of colorectal nodules of mice in different groups (scale bar = 5 mm). F) The nodules were counted based on size. The data are represented as the mean ± SEM of the nodule number. G) Representative H&E‐stained sections of colorectal tissues from each group. H) Blinded histological scoring of the average percentages of adenocarcinomas for mice in each group. I) Immunofluorescence technique for detecting the CD133 protein and exosomes distribution in colorectal tissues from different mice. Immunofluorescence images were representative of six random fields. J) The percentages of GM‐Exo‐positive colorectal tissue cells were analyzed by FCM. The data are represented as the mean ± SEM of each group (*n* = 6) pooled from three independent experiments. *p < 0.05 and **p < 0.01, analyzed by ANOVA.

Next, the roles of GM‐Exo and the S100A9 cargo in CAC tumorigenesis were investigated. A CAC model was induced in BALB/c mice with AOM/DSS, as indicated (**Figure** **5**A). During CAC induction, the mice were treated with GM‐Exo or GM‐Exo^S100A9KD^. GM‐Exo‐treated mice exhibited a greater susceptibility to CAC, as indicated by less weight gain, more colon shortening, more tumor nodules, and more serious colorectal tissue damage (Figure 5B–H). However, the reduction of S100A9 cargo in GM‐Exo partly abrogates susceptibility to CAC (Figure 5B–H). These results demonstrate that GM‐Exo promotes CAC progression in a manner dependent on S100A9. To further observe the roles of GM‐Exo and the S100A9 cargo in the stemness of CAC cells, we detected CD133 levels in colorectal tissues from CAC mice. The results showed that GM‐Exo increased CD133 expression and the percentages of CD133^+^ cells in colorectal tissues (Figure 5I,J). However, GM‐Exo^S100A9KD^ partially decreased CD133 expression and the percentage of CD133^+^ cells (Figure 5I,J), even in the foci that received equal quantities of exogenous exosomes (Figure 5I). Taken together, these results suggest that exosomal S100A9 from G‐MDSCs promotes CAC susceptibility and is involved in enhancing the stemness of cancer cells.

Combining the biological distribution of exogenous GM‐Exo in colorectal tissues with the chemotaxis of MDSCs in response to exosomal S100A9 from MDSCs,^18^ we speculated that the S100A9 cargo may promote the chemotaxis of G‐MDSCs to peripheral blood and colorectal tissues, which suppresses the immune response. The level of S100A9 was higher in intratumoral tissues than in peritumoral tissues (Figure S3A, Supporting Information). In intratumoral tissues, over 90% of MDSCs were CD11b^+^Ly6G^+^ G‐MDSCs, whereas less than 10% of MDSCs were CD11b^+^Ly6C^+^ M‐MDSCs (Figure S3B, Supporting Information). G‐MDSC infiltration into intratumoral tissues of GM‐Exo‐treated mice was increased compared to that in GM‐Exo^S100A9KD−^treated mice (Figure S3C, Supporting Information). Furthermore, we found that the accumulation of G‐MDSCs in the peripheral blood, spleen, and local colorectal tissues in GM‐Exo‐treated CAC mice was increased compared to that in wild‐type (WT) mice and control CAC mice (Figure S3D,E, Supporting Information). The reduction of the S100A9 cargo decreased G‐MDSC accumulation (Figure S3D,E, Supporting Information). The percentage of G‐MDSCs in the bone marrow was unchanged when the mice were treated with GM‐Exo or GM‐Exo^S100A9KD^ (Figure S3D,E, Supporting Information). The deficiency of S100A9 was sufficient to reduce the chemotactic capacity of GM‐Exo (Figure S3F, Supporting Information). In addition, the percentages of total T cells and their subsets in the peripheral blood, as well as the number of CD3^+^CD8^+^ T cells in colorectal tissues, were reduced in GM‐Exo‐treated CAC mice compared to those in control CAC mice (Figure S3H–K, Supporting Information). However, the percentages of total T cells and their subsets in the peripheral blood and the number of CD3^+^CD8^+^ T cells in colorectal tissues were increased in the presence of deficient S100A9 cargo in GM‐Exo (Figure S3H–K, Supporting Information). These data are consistent with the finding that the number of G‐MDSCs in the peripheral blood (Figure S3G, Supporting Information) and colorectal tissues (Figure S3C, Supporting Information) from GM‐Exo‐treated CAC mice was increased compared to that in GM‐Exo^S100A9KD^‐treated CAC mice.

Collectively, GM‐Exo exacerbates colorectal tumorigenesis through S100A9. On the one hand, exosomal S100A9 from G‐MDSCs enhances cancer cell stemness. On the other hand, exosomal S100A9 from G‐MDSCs promotes the chemotaxis of G‐MDSCs to the peripheral blood and colorectal tissue, which weakens antitumor immunity.

### 2.5 Hypoxia Promotes GM‐Exo Production in a HIF‐1α‐Dependent Manner

**Figure 6:**
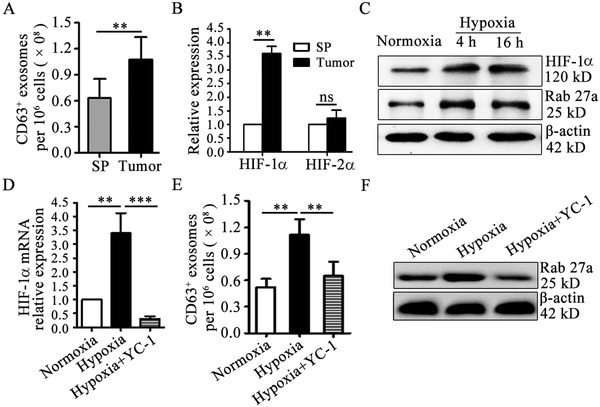
Hypoxia increases GM‐Exo production in a HIF‐1α‐dependent manner. A) Exosomes were quantified from splenic and tumoral G‐MDSCs from CAC mice by ExoELISA. B) The expression of HIF‐1α and HIF‐2α in tumoral G‐MDSCs was evaluated by RT‐qPCR. C) Western blotting was performed to show HIF‐1α and Rab27α protein levels. Splenic G‐MDSCs were cultured at a 1% oxygen (O_2_) concentration in a three‐gas incubator. Representative results from three independent experiments are shown. D) The expression of HIF‐1α in splenic G‐MDSCs was evaluated by RT‐qPCR. In the YC‐1‐treated group, splenic G‐MDSCs were cultured under 1% O_2_ conditions and treated with 50 µmol L^−1^ YC‐1. E) Exosomes were quantified by ExoELISA. F) Western blotting was performed to show Rab27a protein levels. Representative results from three independent experiments. In (A,B) the data are presented as the mean ± SEM of each group pooled from three independent experiments. **p < 0.05, analyzed by a *t*‐test. In (D,E) the data are presented as the mean ± SEM. **p < 0.01 and***p < 0.001, analyzed by ANOVA.

To investigate whether exosomes production from G‐MDSCs is affected by the tumor microenvironment, we first compared exosomes production in splenic and tumoral G‐MDSCs from CAC mice. The results showed that tumoral G‐MDSCs produced more exosomes than did an equal number of splenic G‐MDSCs (**Figure** **6**A). Hypoxia is a common characteristic of solid tumors and plays an important role in the tumor microenvironment. We found more hypoxia‐inducible factor (HIF)‐1α but not HIF‐2α in tumoral G‐MDSCs than in splenic G‐MDSCs (Figure 6B). Next, we analyzed the effect of hypoxia on GM‐Exo production and the role of HIFs by exposing splenic G‐MDSCs to hypoxia. Hypoxia increased HIF‐1α and Rab27a levels in splenic G‐MDSCs (Figure 6C,D). Importantly, hypoxia increased exosomes production from splenic G‐MDSCs (Figure 6E). To observe the role of HIF‐1α in hypoxia‐promoted GM‐Exo production, hypoxia‐treated splenic G‐MDSCs were treated with the HIF‐1α inhibitor 3‐(5′‐hydroxymethyl‐2′‐furyl)‐1‐benzylindazole (YC‐1). The results showed that hypoxia had no effect on exosomes production by splenic G‐MDSCs when HIF‐1α was inhibited (Figure 6D,E). As shown in Figure 6F, the Rab27a level in YC‐1‐treated G‐MDSCs was lower than that in cells exposed only to hypoxia (Figure 6F). Taken together, these data indicate that hypoxia induces G‐MDSCs to produce exosomes in a HIF‐1α‐dependent manner.

### 2.6 Respiratory Hyperoxia Attenuates Colon Cancer Cell Stemness through Inhibiting GM‐Exo Production

**Figure 7:**
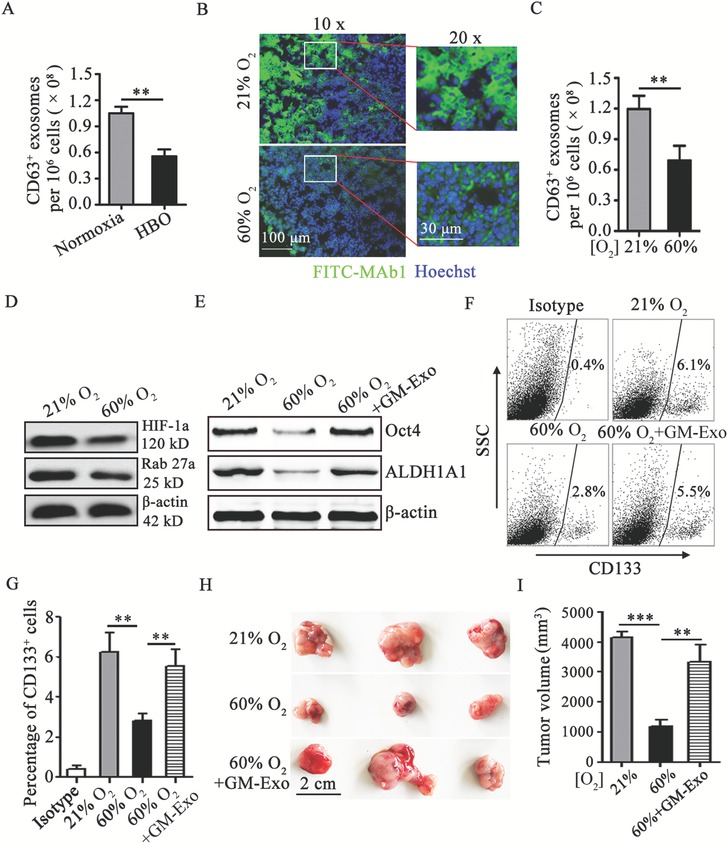
Respiratory hyperoxia attenuates colon cancer cell stemness through inhibiting GM‐Exo production. A) Exosomes were quantified from tumoral G‐MDSCs treated with HBO or maintained at normoxia in vitro by ExoELISA. B) Immunohistochemical demonstration of 60% O_2_ treatment reduce hypoxic areas (green) in tumor tissue. Tissue cryosections from intradermal CT‐26 tumors were prepared, and immunohistochemistry was performed. Immunofluorescence images were representative of six random fields. C) Exosomes were quantified from tumoral G‐MDSCs from CT‐26 bearing mice treated with 60% O_2_ or maintained at 21% O_2_ by ExoELISA. D) Western blotting was performed to show HIF‐1α and Rab27a protein levels in tumoral G‐MDSCs from CT‐26 bearing mice treated with 60% O_2_ or maintained at 21% O_2_. Representative results from three independent experiments. E) Western blotting was performed to show Oct4 and ALDH1A1 protein levels in tumor tissue from CT‐26 bearing mice treated with 60% O_2_ or maintained at 21% O_2_. Representative results from three independent experiments. F,G) The percentage of CD133‐positive tumor tissue cells were analyzed by FCM. The data are represented as the mean ± SEM of each group pooled from three independent experiments. H,I) The effect of respiratory hyperoxia on the development of an CT‐26 cell tumor model. Mice with 15‐day established CT‐26 colon tumors were treated with 60% O_2_, and some of them were treated with 30 µg GM‐Exo via the tail vein once every 3 days from 15‐day. Tumors were harvested at day 25. Representative tumors in each group are shown and the volume of tumors in each group is measured. In A,C) the data are presented as the mean ± SEM of each group pooled from three independent experiments. **p < 0.05, analyzed by a *t*‐test. In G,I) the data are presented as the mean ± SEM. **p < 0.01,***p < 0.001, analyzed by ANOVA.

Taking into account the role of hypoxia in GM‐Exo secretion and the role of GM‐Exo in the stemness of colon cancer cells, we hypothesized that the reduction of tumor hypoxia may inhibit the hypoxia‐driven production of GM‐Exo in the tumor microenvironment and then weak the stemness of colon cancer cells. To test this hypothesis, we first compared the ability of exosomes production in tumoral G‐MDSCs treated with hyperbaric oxygen (HBO) or maintained at normoxia in vitro. The results showed that HBO treated G‐MDSCs produced more exosomes than did an equal number of normoxia treated G‐MDSCs (**Figure** **7**A). Next, CT‐26 cell‐bearing mice were placed in chambers with well‐controlled gas composition (60% O_2_) to mimic protocols of supplemental oxygen delivery to humans.^28^, ^29^ Consistent with the result of previous study from Hatfield et al.,^29^ 60% O_2_ treatment improved significantly hypoxia condition in tumor tissue (Figure 7B). The effect of oxygen treatment on GM‐Exo production and the stemness of colon cancer cells was observed by exposing CT‐26 cell‐bearing mice to 60% O_2_ or maintained at 21% O_2_. The results showed that tumoral G‐MDSCs from mice breathing 60% O_2_ produced more exosomes than did an equal number of G‐MDSCs from mice breathing 21% O_2_ (Figure 7C). 60% O_2_ treatment also decreased HIF‐1α and Rab27a levels in tumoral G‐MDSCs (Figure 7D). Moreover, the percentages of CD133^+^ cells and the expression of Oct4 and ALDH1A1 in 60% O_2_ treated mice were decreased when the mice were treated with 60% O_2_ (Figure 7E–G). Consistent with these results, tumor growth was slower in mice treated with 60% O_2_ compared to mice breathing ambient 21% O_2_ (Figure 7H–I). However, the application of exogenous GM‐Exo could significantly antagonize the effect of 60% O_2_ treatment (Figure 7E–I). These results provide support for the antitumor effect of oxygen treatment was mediated by decreased GM‐Exo secretion.

### 2.7 Exosomal S100A9 from Human MDSCs Promotes Colon Cancer Cell Growth

**Figure 8:**
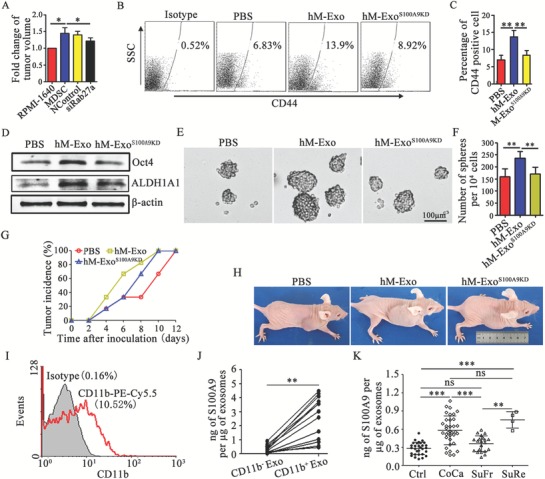
Exosomal S100A9 from human MDSCs promotes colon cancer cell growth. A) Fold change in tumor volume relative to non‐MDSC‐treated SW480 cell‐bearing mice. B,C) Representative percentages of CD44^+^ cells after 10 µg mL^−1^ hM‐Exo treatment were detected by FCM (B), and the results were analyzed statistically (C). D) Western blot analysis was used to determine Oct4 and ALDH1A1 levels after treatment, as indicated. Representative results from three independent experiments. E,F) hM‐Exo promoted SW480 cell sphere formation through S100A9. In the presence of 10 µg mL^−1^ hM‐Exo or hM‐Exo^S100A9KD^, SW480 cells were subjected to the tumor sphere formation assay under sphere‐forming conditions for 14 days. Representative photographs were taken (E), and the number of spheres (F) with a diameter of >75 µm was counted. G,H) The effect of hM‐Exo on the development of an SW480 cell tumor model. The mice were injected with 1 × 10^6^ SW480 cells, which were treated with 10 µg mL^−1^ hM‐Exo for 72 h. The tumor incidence was observed every two days G). Representative mice in each group are shown after 20 days H). I) Representative FCM results for the percentages of CD11b^+^ hM‐Exo. J) CD11b^+^ hM‐Exo were sorted from exosomes by FCM. The S100A9 levels in CD11b^−^ hM‐Exo and CD11b^+^ hM‐Exo from plasma from colon cancer patients. hM‐Exo loaded in the beads were cleaved with RIPA, and S100A9 levels were detected with ELISA. K) Exosomal S100A9 in healthy control subjects (Ctrl), patients with colorectal cancer (CoCa), CRC patients who were tumor free after treatment (SuFr), and CRC patients with tumor relapse after treatment (SuRe). In (A) the data are presented as the mean ± SEM of each group (*n* = 4). *p < 0.05, analyzed by ANOVA. In C,F,K) the data are presented as the mean ± SEM of each group, pooled from three independent experiments. *p < 0.05, **p < 0.01, and ***p < 0.001, analyzed by ANOVA.

Taking into account the roles of exosomal S100A9 from G‐MDSCs in promoting CRC cell stemness and tumorigenesis in CT‐26 cells and CAC mice, we observed whether human exosomal S100A9 from MDSCs had similar characteristics in human colon cancer cells. HLA‐DR^−^CD11b^+^CD33^+^ MDSCs were isolated from tumor tissues from CRC patients. The Rab27a isoform was knocked down with a specific siRNA. MDSCs subjected to Rab27a knockdown and SW480 human colon cancer cells were co‐transferred to BALB/c nude mice. As shown in **Figure** **8**A, human MDSCs promoted SW480 cell growth, but the knockdown of Rab27a in MDSCs diminished tumor growth (Figure 8A). Next, exosomes from human MDSCs (hM‐Exo) was isolated from MDSCs, and the roles of hM‐Exo and the S100A9 cargo in the stemness of cancer cells were observed. The results showed that the percentages of CD44^+^ cells, the expression of stem cell core proteins, and the number of tumor spheres formed were increased when colon cancer cells were treated with hM‐Exo (Figure 8B–F). However, the stemness characteristics of colon cancer cells were decreased when S100A9 was knocked down in hM‐Exo (Figure 8B–F). Furthermore, we established tumor‐bearing mice with hM‐Exo‐treated SW‐480 cells and observed the tumor incidence and progression. Tumorigenesis occurred earlier and tumor growth occurred faster in the hM‐Exo‐treated group than in the hM‐Exo^S100A9KD^‐treated group (Figure 8G,H). These results demonstrate that hM‐Exo enhances the stemness and growth of human colon cancer cells through S100A9.

In addition, we analyzed the percentage of CD11b‐positive exosomes (CD11b^+^ Exo), and sorted CD11b^+^ Exo from plasma from CRC patients. The data showed that ≈10% of plasma exosomes are CD11b^+^ exosomes (Figure 8I). Moreover, the quantity of S100A9 is greater in CD11b^+^ Exo than in CD11b^−^ Exo (Figure 8J). These results suggest that S100A9 is distributed mainly in CD11b^+^ Exo, which may be shed from MDSCs. Therefore, we detected the quantity of plasma exosomal S100A9 in CRC patients. The levels of exosomal S100A9 were higher in CRC patients, and tumor relapse patients exhibit levels higher than those in successful CRC resection patients (Figure 8K). These results suggest that the level of plasma exosomal S100A9 is increased in CRC patients.

## 3 Discussion

The interaction between tumor cells and the host immune system causes immunoediting,^30^ which results in tumor initiation and deterioration. There is increasing evidence for the crosstalk between MDSCs and cancer cells as a new fuel for the cancer deteriorative machinery.^12^, ^31^ The immunosuppressive roles of MDSCs are relatively well‐understood in tumors.^25^, ^32^, ^33^ However, the nonimmunological effects of MDSCs are poorly studied in human CRC. MDSC has been linked to cancer stemness.^12^, ^31^, ^34^ It has been reported that MDSC enhances stem‐like qualities in breast cancer cells.^13^ However, the nature of this interaction is unclear. Exosomes contain proteins and nucleic acid material from their origin cells and have been proposed to act as intercellular communicators between sender cells and receiver cells.^35^, ^36^ Here, we discovered for the first time that a crosstalk between G‐MDSCs and colon cancer cells occurs by exosomes secretion, which conveys stem‐like qualities to cancer cells. In this study, we have generated important novel insights into G‐MDSC and CRC cell communication.

We investigated the key mediator in GM‐Exo that conveyed stem‐like qualities to CRC cells. Unbiased mass spectrometry analysis showed that S100A9 is abundant in MDSC‐derived exosomes protein cargo.^18^ Our results also showed that S100A9 was abundant in GM‐Exo. The upregulated S100A9 expression in MC‐38 cells could promote invasion and migration,^37^ which are related to cancer stemness. These results led us to identify S100A9 as a candidate protein. In fact, the tumor sphere formation ability was decreased, and the levels of a panel of established CSC markers, including CD133, CD44, Sox2, Oct4, Nanog, and ALDH1A1, were decreased in colon cancer cells treated with GM‐Exo subjected to S100A9 knockdown. Moreover, exogenous GM‐Exo promoted cancer cell stemness via S100A9 in vivo, which enhanced the susceptibility of mice to AOM/DSS‐induced CAC. Here, we demonstrate a novel role for exosomal S100A9 from G‐MDSCs in CRC cell stemness. S100A9 directly binds to components of the NADPH oxidase complex in myeloid cells. This binding complex potentiates the activation of NADPH oxidase. NADPH oxidase is the major source of ROS in biological systems.^25^, ^38^ Consistent with this process is the observation that the levels of Nox4 and ROS in cancer cells are enhanced after GM‐Exo treatment. Such oxidative stress conditions facilitate tumor growth in multiple ways by causing DNA damage and genomic instability and ultimately, by reprogramming cancer cell metabolism.^39^ Zhang et al. showed that the stemness of pancreatic cancer cells was promoted through the Nox/ROS/NF‐κB/STAT3 signaling cascade.^26^ The S100A9 protein in exosomes from chronic lymphocytic leukemia cells promotes NF‐κB activity during disease progression.^40^ Our results also showed that S100A9 enhanced the phosphorylation of STAT3 and NF‐κB in colon cancer cells. Collectively, these results suggest that exosomal S100A9 from G‐MDSCs promotes the stemness of cancer cells and the subsequent development of CRC.

S100A8/A9 bind to carboxylated N‐glycans expressed on the receptor for advanced glycation end‐products (RAGE) and other cell surface glycoprotein receptors on MDSCs, signal through the NF‐κB pathway, and promote MDSC migration.^41^ Our results demonstrated that GM‐Exo was chemotactic for G‐MDSCs, and chemotaxis was significantly inhibited in S100A9‐deficient GM‐Exo‐treated G‐MDSCs in vitro. This result is consistent with the findings of Burke et al..^18^ In vivo, we found that exosomal S100A9 from G‐MDSCs promoted the accumulation of G‐MDSCs in the peripheral blood and intratumoral tissues during colorectal carcinogenesis. These results improve our understanding of the reason for MDSC accumulation, although the mechanism by which exosomal S100A9 from G‐MDSCs promotes G‐MDSC migration needs to be further clarified. Several factors have been implicated in the T cell‐suppressive activities of G‐MDSCs.^42^, ^43^ We observed that T cells and their subpopulations were decreased when tumor‐bearing mice were treated with GM‐Exo, which suggests that the antitumor response is inhibited. These results indicate that GM‐Exo mediate the chemotaxis of G‐MDSCs through their S100A9 content and participate in immunosuppression.

There is considerable evidence that the number of exosomes shed by cancer cells increases with disease progression.^44^ Intratumoral hypoxia may alter the behavior of the bulk population.^45^ In our study, the number of exosomes shed by tumoral G‐MDSCs was greater than that shed by splenic G‐MDSCs, but the internal causes promoting the production of exosomes by tumoral G‐MDSCs have not been delineated. We demonstrated that hypoxia, a critical microenvironmental stimulus in CRC, stimulated HIF‐1α‐dependent Rab27a expression and increased exosomes production. However, further studies are required to determine whether there are qualitative differences in the S100A9 cargo between hypoxic and normoxic G‐MDSCs. The exposure of splenic G‐MDSCs to hypoxia significantly increased HIF‐1α levels and exosomes production. Moreover, the inhibition of HIF‐1α could downregulate Rab27a expression and exosomes production during hypoxia. However, the molecular mechanism by which HIF‐1α regulates Rab27a expression and exosomes production remains unclear. These preclinical data provide a scientific foundation for further trials that test the effect of oxygen treatment on the blockade of exosomes production from tumoral G‐MDSCs and the inhibition of colon cancer cells stemness. In accordance with previous study focused on the antitumor effects of 60% O_2_,^29^ our results showed that oxygen treatment inhibited tumor growth. Moreover, this antitumor effect could be accounted for by the inhibition of GM‐Exo‐mediated stemness of colon cancer cells. This is another important potential strategy of the inhibition of tumor growth by oxygen treatment.

Our functional experiments in mouse cell lines and murine models drove us to detect the role of human MDSC‐shed exosomes in human CRC stemness. We found that MDSCs promoted CRC cell growth through exosomes production. Additionally, exosomes shed by tumoral MDSCs from CRC patients enhanced the stemness of CRC cells through S100A9 and sequential tumor progression. Therefore, exosomal S100A9 from human MDSCs also enhanced human CRC cell stemness. These findings prompted us to investigate the potential value of exosomal S100A9 from MDSCs for CRC. Previous research showed that CD11b is present on the surface of MDSC exosomes.^46^ Here, we compared the difference in S100A9 levels between CD11b^+^ Exo and CD11b^−^ Exo. The results showed that the quantity of S100A9 was substantially greater in CD11b^+^ Exo than in CD11b^−^ Exo. These results suggested that these S100A9‐abundant exosomes were derived from MDSCs. Moreover, the level of plasma exosomal S100A9 in CRC patients was higher than that in healthy subjects, and the levels of plasma exosomal S100A9 in postoperative recurrence patients were higher than those in postoperative patients without recurrence. Overall, our results suggest that the level of plasma exosomal S100A9 has potential value for CRC, although much work remains to be completed.

## 4 Conclusion

In summary, our findings reveal that G‐MDSCs enhance CRC cell stemness via exosomes and exosomal S100A9 in the tumor microenvironment, especially under hypoxic conditions. Respiratory hyperoxia could reduce the stemness of colon cancer cells through inhibiting GM‐Exo production. High levels of plasma exosomal S100A9 is associated with CRC occurrence and recurrence. Block MDSC exosomes production and the related component may provide a novel approach for CRC therapy.

## 5 Experimental Section

*Cell Lines and Mice*: The murine CRC cell line CT‐26 and the human CRC cell line SW480 were purchased from the American Type Culture Collection. All cells were maintained in a humidified incubator with 5% CO_2_ at 37 °C. Female BALB/c mice and BALB/c nude mice were from the Animal Research Center of Jiangsu University and the Animal Research Center of Yangzhou University, respectively. All mice were housed in a specific pathogen‐free animal facility and used at 6–8 weeks of age. All animal experiments were carried out in accordance with a protocol approved by the Jiangsu University Animal Ethics and Experimentation Committee.

*Building a Transplantation Model of Colon Tumors and Treatment*: CT‐26 cell‐ or SW480 cell‐bearing mice were established with CT‐26 cells and SW480 cells, respectively, in BALB/c mice or BALB/c nude mice, similar to a method previously used in the laboratory.^47^ Briefly, the mice were subcutaneously injected in the right flank with 1 × 10^6^ tumor cells. When the role of exosomes was observed in MDSC‐mediated tumor progression, murine G‐MDSCs and M‐MDSCs or human MDSCs were transfected with Rab27a siRNA; 1 × 10^6^ G‐MDSCs or M‐MDSCs were then transferred to CT‐26 cell‐bearing mice in the tumor area once every 7 days starting the first day, and 1 × 10^6^ human MDSCs were transferred to BALB/c nude mice with 1 × 10^6^ SW480 cells on the first day. When the role of GM‐Exo was observed directly in promoting CT‐26 cell stemness, CT‐26 cells were treated with 10 µg mL^−1^ GM‐Exo for 72 h before being injected into BALB/c mice. When the role of oxygen treatment was observed in inhibiting tumor growth, CT‐26 cell‐bearing mice were placed in chambers with well‐controlled gas composition to mimic protocols of supplemental oxygen delivery to humans.^28^, ^29^ Confirmation of the levels of O_2_ in control and experimental groups treated with 21% or 60% O_2_ was done. Mice with 15‐day established CT‐26 colon tumors were treated with 60% O_2_ for 1 h every day or maintained at 21% O_2_. Among 60% O_2_ treated mice, some were treated with 30 µg of GM‐Exo in 100 µL of PBS via the tail vein once every 3 days from 10‐day. All tumors were harvested at day 25. When the role of human MDSC‐derived exosomes (hM‐Exo) was directly observed in promoting SW480 cell stemness, SW480 cells were treated with 10 µg mL^−1^ exosomes for 72 h before being injected into BALB/c nude mice. Tumor length (*L*), diameter and width (*W*) were measured with a caliper. Tumor volumes were calculated using the formula *V* = π × *L* × *W* ^2^/6.

*Building a CAC Model and Treatment*: BALB/c mice were given a single intraperitoneal injection of AOM (10 mg kg^−1^ body weight) at the age of 8 weeks. Starting 1 week after the injection, the animals received 2% DSS in their drinking water for 7 days and then no further treatment for 14 days. A cycle is 21 days. The mice were exposed to five cycles throughout the process. During this process, the mice were treated with 100 µL of phosphate‐buffered saline (PBS) (A/D group), 30 µg of GM‐Exo in 100 µL of PBS (A/D + GM‐Exo group), or 30 µg of GM‐Exo^S100A9KD^ in 100 µL of PBS (A/D + GM‐Exo^S100A9KD^ group) via the tail vein once every 7 days from the first cycle. When the exosomes distribution was observed, the exosomes were labeled with PKH67 or PKH26. Wild‐type mice that were treated with PBS (WT group) served as a blank control group. The animals were constantly monitored for body weight.

All animals were sacrificed at the end of the study by bloodletting followed by cervical dislocation. Whole blood was collected with blood collection tubes that contained EDTA‐Na_2_. The fur and cheekbones were removed and washed with PBS. The bone marrow cells were collected, and red blood cells were removed by ACK lysis buffer.

The lengths of the large bowels (from the ileocecal junction to the anal verge) were measured. The large bowels were cut open longitudinally along the main axis and then washed with saline. The number of tumor nodules was counted based on size. The large bowel was cut and fixed in 10% buffered formalin for at least 24 h. Histological examination was performed on paraffin‐embedded sections after H&E staining.

When the S100A9 levels in colorectal tissue was observed with immunohistochemistry (IHC), the sections from the formalin fixed, paraffin‐embedded tissues were deparaffinized and rehydrated. Then, the sections were boiled for 10 min in 0.01 m citrate buffer and incubated with 0.3% H_2_O_2_ in methanol to block endogenous peroxidase. In addition, the sections were incubated with the anti‐S100A9 antibody (10 µg mL^−1^), followed by incubation with a secondary antibody tagged with the peroxidase enzyme. The sections were visualized with 0.05% DAB until the desired brown reaction product was obtained. All slides were observed under a Nikon E400 light microscope, and representative photographs were taken.

When the exosomes distribution and CD133 levels were observed in colorectal tissue with immunofluorescence, tissue cryosections were blocked with 0.5% BSA for 1 h and stained with a PE‐conjugated antibody against CD133 (1:100, Miltenyi Biotec). The nuclei were stained with Hoechst 33 342. Fluorescence images were obtained using a Nikon microscope and analyzed using Nikon software. When exosomes uptake was observed, cell suspensions of colorectal tissues (the preparation method is detailed below) were prepared, and the percentage of PKH67^+^ cells was detected with flow cytometry (FCM).

*MDSC Isolation*: Murine MDSCs, G‐MDSCs, and M‐MDSCs were isolated from the spleens of CT‐26 tumor‐bearing mice or CAC mice using a mouse MDSC isolation kit (Miltenyi Biotec, Auburn, CA) according to the manufacturer's instructions.^47^, ^48^ The immunosuppression of splenic G‐MDSCs was detected by a T cell proliferation assay. For human MDSC or murine G‐MDSC isolation from tumor tissues from CT‐26 cell‐bearing mice or CAC mice or CRC patients, quantitative solid tumors and paracancerous tissues were dissected and mechanically dissociated into small, <2 mm^3^ fragments with a scalpel, followed by digestion with digestion solution containing 0.5 mg mL^−1^ collagenase type І, 0.2 mg mL^−1^ hyaluronidase, and 0.15 mg mL^−1^ DNase for 2 h at 37 °C on a rotating platform to obtain single‐cell suspensions. Red blood cells were removed by ACK lysis buffer. Murine G‐MDSCs were further isolated by an MDSC isolation kit. Human MDSCs were harvested with FCM.

*Exosomes Purification, Characterization, and Analyses*: GM‐Exo, Neu‐Exo, and hM‐Exo were isolated using a procedure similar to that described in the previous report.^20^ Cells were removed from culture supernatants by centrifugation at 500 g for 5 min. To remove any possible apoptotic bodies and large cell debris, the supernatants were then centrifuged at 300 g for 20 min and 10 000 g for 30 min. Finally, exosomes were collected by an exosomes extraction kit. The final exosomes pellet was resuspended in PBS, and the protein concentration was detected with a Micro BCA protein assay kit, which was from Beijing ComWin Biotech (Beijing, China). Electron micrographs were observed by transmission electron microscopy (Tecnai‐12; Philips, Amsterdam, Netherlands). Exosomes were lysed, and CD63, CD9 or calnexin was detected by western blotting.

The immunosuppression of exosomes was detected by a T cell proliferation assay. During the purification process of GM‐Exo^S100A9KD^ or hM‐Exo^S100A9KD^, in which S100A9 expression is knocked down, murine G‐MDSCs and human MDSCs that were used to extract exosomes were plated in 24‐well plates and then transfected with 50 × 10^−9^ m S100A9 siRNA or its negative control (RiboBio Co., Guangzhou, China) following the manufacturer's protocol.

*T Cell Proliferation Assay*: This experiment was performed according to the previous report.^49^

*ELISA*: For the detection of IFN‐γ, CD4^+^ T cell culture supernatants were collected. The IFN‐γ contents in the supernatant were detected with the mouse IFN‐γ Sunny ELISA Assay kit (Multi Sciences, Hangzhou, China) following the manufacturer's instructions. For the detection of exosomal S100A9, exosomes were isolated from CRC patient plasma and lysed. The S100A9 level in exosomes was detected with a human S100A9 ELISA Assay kit from Biofine (Shanghai, China), using 2 µg of exosomes per 100 µL of sample diluent, in duplicate reactions, according to the manufacturer's instructions.

*Small Interfering RNA (siRNA) Treatment*: MDSCs or their subpopulations were plated in 24‐well plates and then transfected with 100 × 10^−9^ m Rab27a, S100A9 siRNA or their negative controls (RiboBio Co) following the manufacturer's protocols.

*Exosomes Labeling and Detection*: For exosomes‐tracking purposes, purified exosomes were fluorescently labeled using the PKH67 (green) or PKH26 (red) membrane dye (Sigma‐Aldrich) according to the manufacturer's instructions. Labeled exosomes were washed in 20 mL of PBS, collected by ultracentrifugation and resuspended in PBS. To measure exosomes uptake by CT‐26 cells, labeled exosomes were added to the culture medium, and images were obtained for exosomes‐positive cells by immunofluorescence. Pictures were taken at a 40 × magnification. CT‐26 cells were collected and washed with PBS, and exosomes uptake by CT‐26 cells was analyzed with imaging flow cytometry. To observe the distribution of exosomes in the colorectal tissue, 30 µg of total exosomal protein was injected via the tail vein in a total volume of 100 µL PBS at the end of modeling. Colorectal tissues were isolated after 24 h, and paraffin sections were made. The distribution of exosomes was observed by immunofluorescence.

*Colony Formation Assay*: Five hundred CT‐26 cells were seeded into petri dishes with a diameter of 30 mm and incubated at 37 °C in 5% CO_2_ for 14 days. The medium was changed at 2‐day intervals. At the end of the incubation period, the cultures were fixed with 4% paraformaldehyde and stained with crystal violet. The number of colonies was counted.

*G‐MDSC Chemotaxis*: First, 500 µL of fresh medium or medium from G‐MDSC cultures (conditioned medium) or 30 µg mL^−1^ GM‐Exo or GM‐Exo^S100A9KD^ in fresh medium was placed in individual wells of 24‐well plates (lower compartment) were included in some wells. The concentration of purified exosomes was equivalent to the concentration of exosomes in conditioned medium. Transwells with an 8 µm polycarbonate semipermeable membrane were inserted in each well, and 1 × 10^6^ splenic G‐MDSCs from CAC mice in 100 µL of serum‐free RPMI 1640 medium were placed in the transwells (upper compartment). The assembled transwells were incubated at 37 °C in 5% CO_2_ for 6 h, and the G‐MDSCs in the bottom chamber were then quantified.

*Flow Cytometry*: For analysis of tumor cell lines or splenic and tumoral single‐cell suspensions, cells were prepared by filtering through a 70 µm strainer before incubation with a fluorescent antibody. For blood analysis, blood samples were collected, and erythrocytes were lysed by ACK lysis buffer. The cells were analyzed using the fluorescence‐conjugated antibodies. Murine G‐MDSCs were analyzed using a PE‐conjugated anti‐Ly‐6G mAb (RB6‐8C5) and a FITC‐conjugated anti‐CD11b mAb (M1/70), which were purchased from Biolegend (San Diego, CA, USA). Murine M‐MDSCs were analyzed using a PE‐conjugated anti‐Ly‐6C mAb (RB6‐8C5) and a FITC‐conjugated anti‐CD11b mAb, which were purchased from Biolegend. Human MDSCs were analyzed and sorted using a FITC‐conjugated anti‐CD11b mAb (M1/70), an APC‐conjugated anti‐33 mAb (HIM3–4), and a PE‐conjugated anti‐HLA‐DR mAb (LN3), which were purchased from Biolegend. Cells that combined with PKH67‐exosomes were analyzed. Cells that combined with PKH26‐exosomes were analyzed. The stemness of CT‐26 cells was analyzed using a PE‐conjugated anti‐CD133 mAb (AC133) and a FITC‐conjugated anti‐CD44 mAb (DF1485) from Miltenyi Biotec. The stemness of SW480 cells was analyzed using a FITC‐conjugated anti‐CD44 mAb (DF1485) from Miltenyi Biotec. Murine T cells were analyzed with a PE‐conjugated anti‐CD3‐PE mAb (LT3), a PE‐CY5.5‐conjugated anti‐CD4 mAb (L3T4), and a FITC‐conjugated anti‐IFN‐γ‐ mAb (XMG1.2), which were from eBioscience (San Diego, CA, USA). For the detection of ROS, CT‐26 cells in 1 mL of PBS were treated with 30 ng mL^−1^ phorbol‐12‐myristate‐13‐acetate (PMA) and 2.5 × 10^−9^ m H_2_DCFDA for 30 min, and fluorescence was analyzed. Flow cytometry was performed using FACSCalibur Flow Cytometer (Becton Dickinson).

*Western Blot Analysis*: Exosomes or cells were lysed with radio immunoprecipitation assay (RIPA) buffer containing a complete protease inhibitor tablet (Roche). The lysates were cleared by centrifugation at 14 000 g for 20 min. The protein concentrations of the supernatant fractions were detected with a Micro BCA protein assay kit. Then, 50 µg of lysate was electrophoresed via 12% sodium dodecyl sulfate‐polyacrylamide gel electrophoresis (SDS‐PAGE) and subsequently electrotransferred onto immobilon polyvinylidene membranes (Bio‐Rad, CA, USA) followed by 1 h of blocking and primary antibody incubation. The samples were immunoblotted overnight at 4 °C with the following primary antibodies: CD9 (1:1000), CD63 (1:1000), calnexin (1:1000), S100A9 (1:1000), p‐STAT3 (1:1000), t‐STAT3 (1:10000), t‐NF‐κB/p65 (1:1000), p‐p65 (1:1000), and β‐actin (1:1000, Abcam); Sox2 (1:800), Oct4 (1:800), Nanog (1:800), ALDH1A1 (1:800), Nox4 (1:800), and Rab27a (1:800, Abways);and HIF‐1α (1:1000, CST). A horseradish peroxidase (HRP)‐linked anti‐rabbit IgG antibody (1:8000, CST) and an HRP‐linked anti‐mouse IgG antibody (1:3000, CST) were used as secondary antibodies.

*RNA Isolation and Quantitative Real‐Time PCR*: Total RNA was extracted from the samples with TRIzol reagent (Invitrogen, CA, USA), according to the manufacturer's instructions. The cDNA was synthesized with random primers and the ReverTra Ace qPCR RT kit (Toyobo, Osaka, Japan). Real‐time PCR was performed in duplicate using Bio‐Rad SYBR Green Super Mix. The primer sequences were as follows: mouse HIF‐1α, 5′‐AGCCCTAGATGGCTTTGTGA‐3′ (forward), 5′‐TATCGAGGCTGTGTCGACTG‐3′ (reverse); mouse HIF‐2α: 5′‐CTATGGACGGCGAGGACTTC‐3′ (forward), 5′‐CTGGAAGATGCTGGTCATGG‐3′ (reverse); mouse β‐actin: 5′‐TGGAATCCTGTGGCATCCATGAAAC‐3′ (forward), 5′‐TAAAACGCAGCTCAGTAACAGTCCG‐3′ (reverse). The level of each gene was expressed as the ratio to the β‐actin transcript level. The data were analyzed by Bio‐Rad CFX Manager software. Relative expression was calculated by using the comparative *C*_t_ method (2^−Δ^ *^C^* ^t^). Primer sequences are available upon request.

*Spheroid Formation Assay*: For the spheroid formation assay, 80% confluent cultures were harvested with trypsin and gently pipetted to form a single CT‐26 cell or SW480 cell suspension. Trypsin was inactivated by the addition of serum‐containing medium, and the cells were collected by centrifugation at 800 rpm for 5 min. The cells were resuspended in spheroid medium, which was serum‐free RPMI‐1640 medium (Gibco, USA) containing 20 ng mL^−1^ epidermal growth factor (EGF) (PeproTech Inc., Rocky Hill), 10 ng mL^−1^ basic fibroblast growth factor (FGF) (PeproTech Inc) and 10 µg mL^−1^ B27 supplement (Gibco), and plated at 40 000 cells per 9.5 cm^2^ well (Corning, USA). When the effect of exosomes was observed, 2 × 10^6^ G‐MDSCs/M‐MDSCs or Rab27a siRNA‐treated G‐MDSCs/M‐MDSCs were added to each well when the medium was changed. When the effect of exosomal S100A9 was observed, 10 µg mL^−1^ exosomes or exosomes^S100A9KD^ were maintained in each well. The cells were cultured for 14 days, and half of the medium was replaced every two or three days. Spheroids with a diameter >75 µm were counted under a light microscope.

*Human or Mouse Plasma Preparation and Colorectal Tissue Collection*: Human peripheral blood samples were obtained from CRC patients and healthy controls at the Affiliated People's Hospital, Jiangsu University. Blood samples were collected in EDTA‐coated tubes and allowed to sit at room temperature for 30 min. The blood was then centrifuged at 1200 g for 10 min at 4 °C to separate the plasma. The plasma was transferred to a clean tube, centrifuged again at 1800 g for 10 min at 4 °C, and stored at −80 °C.^50^ Human CRC tissues were obtained from Jiangsu University Affiliated Hospital, and all cases were pathologically confirmed. Murine blood was obtained from tumor‐bearing mice with EDTA‐coated tubes and allowed sit at room temperature for 30 min. Whole blood was centrifuged at 3000 g for 20 min at 4 °C to separate plasma, which was stored at −80 °C. The study was approved by the Institutional Ethics Committee of Affiliated People's Hospital, Jiangsu University. All individuals provided written, informed consent for blood or tissue donation.

*Hyperbaric Oxygen Treatment*: Tumoral G‐MDSCs from CT‐26 cell‐bearing mice were plated in 24‐well plates within exosomes‐free RPMI‐1640 medium. The medical oxygen chamber is disinfected with UV rays and filled with pure oxygen. 24‐well plates were placed in the oxygen chamber. The pressure was adjusted slowly to 0.2 MPa and maintained for 30 min. Then the 24‐well plates were moved out from the oxygen chamber and cultured for 19 h under normoxia.

*Evaluation of Tumor Microenvironment (TME) Hypoxia*: At day 25, mice in 60% oxygen or maintained at 21% oxygen were injected with hypoxyprobe‐1 (60 mg kg^−1^) via the tail vein. After 3 h of labeling, tumors were snap‐frozen, 5 µm cryosections were prepared and blocked with 0.5% BSA for 1 h and stained with a FITC‐conjugated antibody against hypoxyprobe‐1 (1:100, Hypoxyprobe, Inc). The nuclei were stained with Hoechst 33 342. Fluorescence images were obtained using a Nikon microscope and analyzed using Nikon software.

*Exosomes Isolation from Human or Mouse Plasma*: Exosomes were isolated according to an existing report.^50^ Plasma samples were thawed on ice and centrifuged at 1500 g for 10 min at 4 °C. The supernatants were collected and centrifuged at 10 000 g for 20 min at 4 °C to remove large vesicles. Thrombin (System Bioscience, TMEXO‐1) was added to the supernatant (v:v = 1:100) and incubated for 5 min at room temperature. The supernatants were centrifuged at 10 000 g for 5 min to remove fibrinogen. The supernatants were incubated with ExoQuick for 60 min at 4 °C (v:v = 5:1). The ExoQuick supernatant sample was then centrifuged twice at 1500 g for 30 and 5 min, respectively. The pellet was resuspended in 50 µL of PBS. The protein contents of exosomes were quantified using a Micro BCA protein assay kit.

*CD11b‐Positive Exosomes Sorting by Flow Cytometry (FCM)*: Prepared plasma exosomes were incubated with latex/aldehyde beads at 37 °C for 1 h, and the beads were then washed with 10 mL of PBS and centrifuged at 300 g for 20 min at 4 °C. Then, 2 µg of PE‐conjugated anti‐human CD11b mAb was added to the suspension of beads and incubated at 4 °C for 30 min. CD11b^+^ beads were sorted by flow cytometry, and CD11b^−^ beads were also collected. The beads were centrifuged at 300 g for 20 min at 4 °C. Bead‐loaded exosomes were lysed with RIPA buffer and centrifuged at 300 g for 20 min at 4 °C. The supernatants were collected, and protein concentrations were measured using a Micro BCA protein assay kit.

*Exosomes Quantification by ExoELISA*: Exosomes particles were quantified with an ExoELISA complete kit (CD63 detection) (SBI) according to the manufacturer's instructions.

*Statistical Analysis*: Error bars for graphical data represent the mean ± SEM. Mouse experiments were performed in duplicate or triplicate, using 3–6 mice per treatment group. Statistical significance was determined by a *t*‐test and one‐way ANOVA using GraphPad Prism 6.0 software. Statistical significance was defined as a *p*‐value < 0.05.

## Conflict of Interest

The authors declare no conflict of interest.

## Supporting information

Supplementary
