# Supplementary material for: Granulocytic Myeloid‐Derived Suppressor Cells Promote the Stemness of Colorectal Cancer Cells through Exosomal S100A9
Source: Adv Sci (Weinh). 2019 Jul 22;6(18):1901278. doi: 10.1002/advs.201901278 (PMC6755519; doi:10.1002/advs.201901278)
Supplement: Supplementary file 1 — Supplementary [file ADVS-6-1901278-s001.pdf]

Copyright WILEY-VCH Verlag GmbH & Co. KGaA, 69469 Weinheim, Germany, 2019.

ADVANCED SCIENCE
Open Access

Supporting Information

for *Adv. Sci.*, DOI: 10.1002/advs.201901278

Granulocytic Myeloid-Derived Suppressor Cells Promote the Stemness of Colorectal Cancer Cells through Exosomal S100A9

*Yungang Wang, Kai Yin, Jie Tian, Xueli Xia, Jie Ma, Xinyi Tang, Huaxi Xu, and Shengjun Wang**

Supporting Information

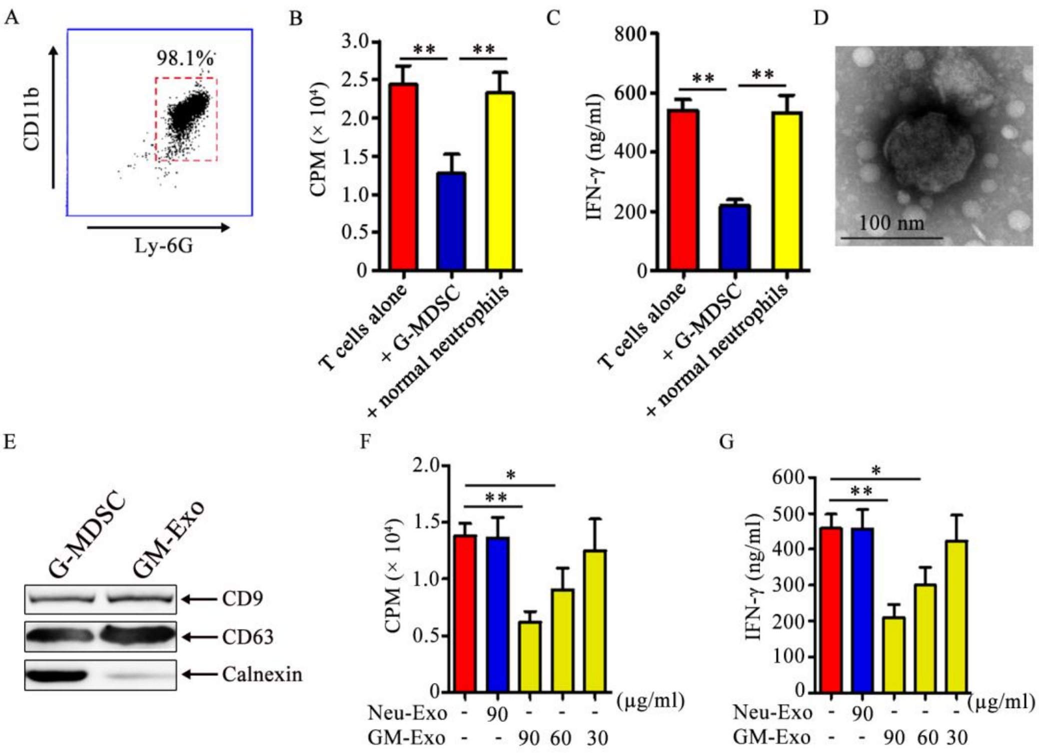

Supplementary figure 1. Extraction and identification of GM-Exo. **A,** Representative micrograph of CT-26 cells (magnification, 200×). **B,** Representative image of CT-26 tumor-bearing mice. **C,** Immunomagnetic beads were used to sort G-MDSCs from the spleens of CT-26-bearing mice. Ly-6G and CD11b were analyzed by FCM. **D,** G-MDSCs suppressed $CD4^+$ T cell proliferation. The counts per min (CPM) values of various wells were detected with a $[^3H]$-thymidine incorporation experiment after the coculture of $CD4^+$ T cells and G-MDSCs. **E,** G-MDSCs suppressed IFN-γ secretion from $CD4^+$ T cells. The IFN-γ contents in the culture supernatants were detected by ELISA. **F,** Representative transmission electron micrograph of GM-Exo (magnification, 135,000×). **G,** The CD9, CD63, and calnexin expression levels of GM-Exo were detected by western blotting. **H,** GM-Exo suppressed $CD4^+$ T cell proliferation. The CPM values of various wells were detected with a $[^3H]$-thymidine incorporation experiment after the culture of $CD4^+$ T cells in the presence of GM-Exo. **I,** GM-Exo suppress IFN-γ secretion from $CD4^+$ T cells. The IFN-γ contents in culture supernatants were detected by ELISA. The data shown in **A**, **B**, **C**, **F**, and **G** were from one of three independent experiments. The data are shown as the mean ± SEM of each group (n = 3) pooled from three independent experiments. *p < 0.001, **p < 0.01, analyzed by ANOVA.

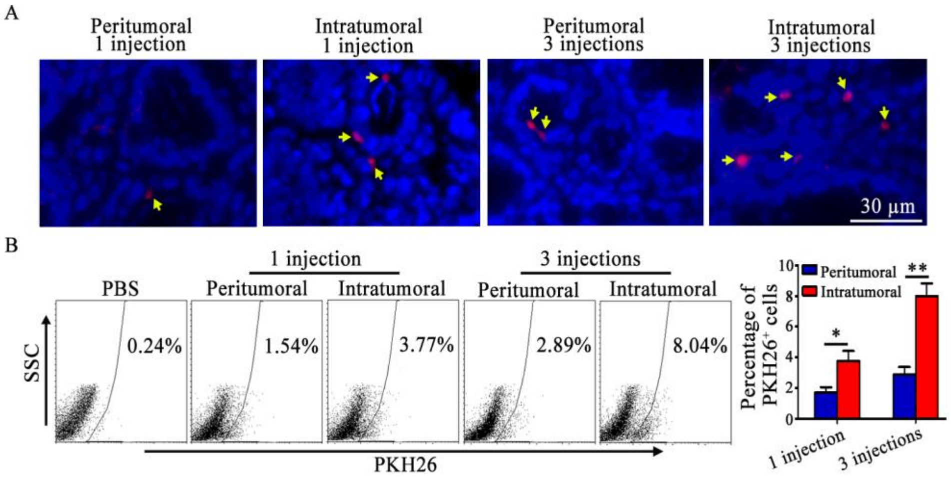

Supplementary figure 2. Biodistribution of exogenous GM-Exo in colorectal tissues from CAC mice. **A,** Images of colorectal tissues from fluorescence microscopy. The arrows indicate exosomes foci. The immunofluorescence images are representative of six random fields. **B,** The percentage of GM-Exo-positive colorectal tissue cells was quantitated by FCM (n = 6). The data are shown as the mean ± SEM of each group pooled from three independent experiments.* $p < 0.05$; ** $p < 0.01$, analyzed by a t-test.

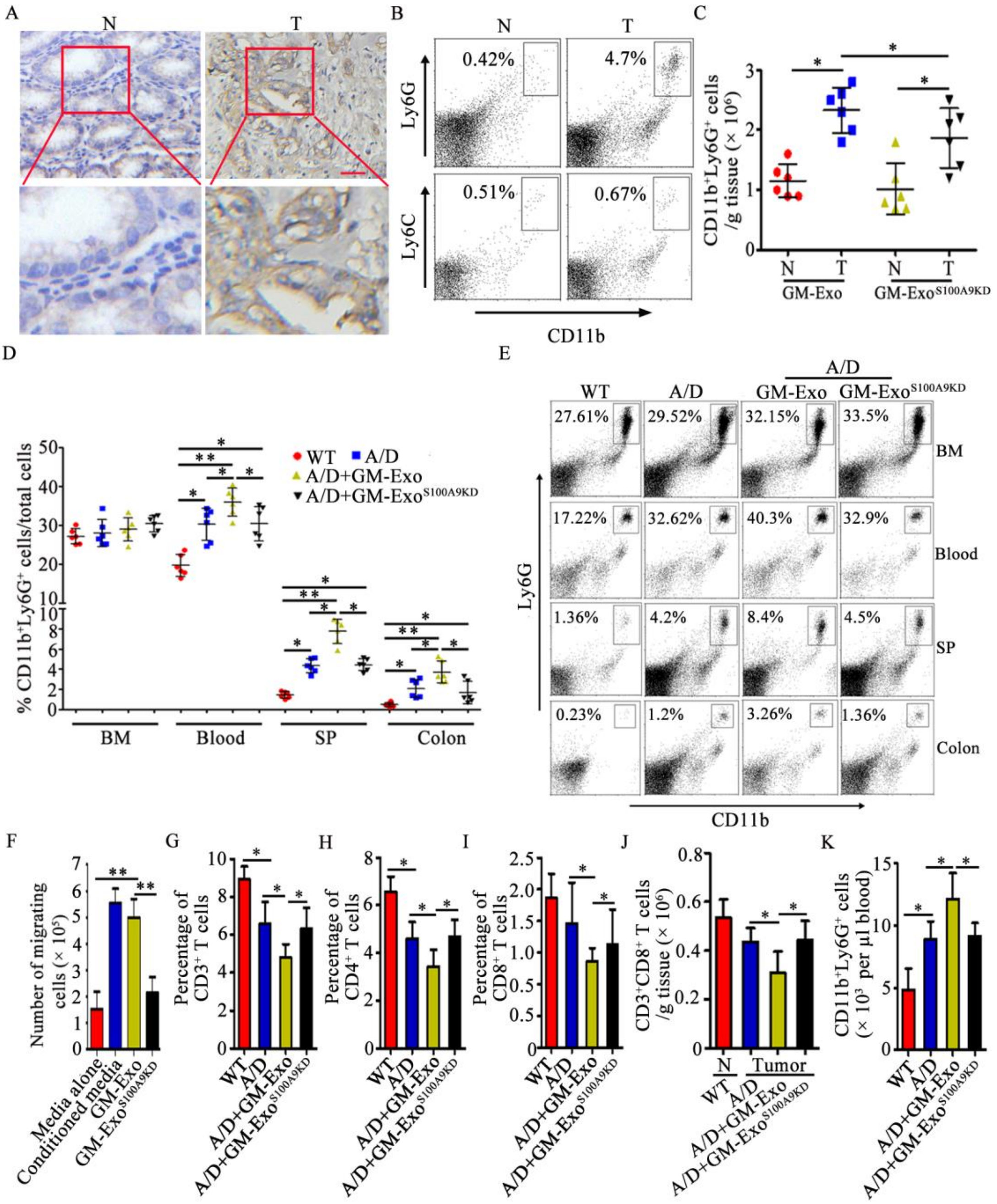

Supplementary figure 3. Exosomal S100A9 from G-MDSCs participates in immunosuppression through mobilizing G-MDSCs to the peripheral blood and colorectal tissues. **A,** Representative images of immunohistochemical staining for S100A9 in tumors (T) and adjacent tissues (N) are shown. Scale bar = 100 μm. **B,** Representative results for the percentages of MDSC subpopulations in peritumoral (N) and intratumoral (T) colorectal tissues from CAC mice. **C,** The quantity of G-MDSCs in colorectal tissues was represented as the number of G-MDSCs per gram. **D** and **E**, The percentages of G-MDSCs in the bone marrow (BM), peripheral blood (PB), spleen (SP), and colorectal tissues of mice in different groups were analyzed by FCM (**D**). Representative diagrams are shown (**E**). **F,** Exosomal S100A9 from G-MDSCs was chemotactic for G-MDSCs. G-MDSCs were placed in the upper compartment of a transwell, and tumor-conditioned medium, GM-Exo or GM-Exo^{S100A9KD} were placed in the lower compartment. The number of G-MDSCs migrating to the lower compartment was determined. **G,** G-MDSC quantification in mouse blood

from different groups (n=6). **H-J,** The percentages of T cells and subsets in mouse blood (n = 6) from different groups were determined by FCM. **K,** The numbers of $CD3^{+}CD8^{+}$ T cells in colorectal tissues from mice in different groups. The data are presented as the mean ± SEM of each group pooled from three independent experiments. *$p < 0.05$, **$p < 0.01$, analyzed by ANOVA.
